# Hybrid Deep Learning–Geostatistical Mapping of Forest Aboveground Biomass in Lishui, China

**DOI:** 10.3390/plants15040587

**Published:** 2026-02-12

**Authors:** Rui Qian, Qilin Zhang, Yuying Gong, Jingyi Wang, Xiaolei Cui, Xiong Yin, Mingshi Li

**Affiliations:** Co-Innovation Center for Sustainable Forestry in Southern China, College of Forestry, Nanjing Forestry University, Nanjing 210037, China; qianrui01@njfu.edu.cn (R.Q.); zhangqilin@njfu.edu.cn (Q.Z.); gongyuying@njfu.edu.cn (Y.G.); wangjingyi@njfu.edu.cn (J.W.); cuixiaolei@njfu.edu.cn (X.C.); yinxiong@njfu.edu.cn (X.Y.)

**Keywords:** forest aboveground biomass, CNN-Transformer, Sentinel-2, ALOS PALSAR, geostatistics, Random Forest

## Abstract

Forest aboveground biomass (AGB) is a key indicator of forest productivity and carbon sequestration, yet many remote sensing AGB models overlook spatial autocorrelation in plot observations and model residuals. This study proposes a hybrid framework that combines a CNN-Transformer (Convolutional Neural Network-Transformer) model with geostatistical Kriging of residuals to improve regional AGB mapping in Lishui City, Zhejiang Province, China. Using 398 forest plots and multi-source predictors derived from Sentinel-2 imagery, ALOS-2 PALSAR-2 SAR data, and ALOS 12.5 m DEM, relevant variables were screened using Random Forest importance ranking. The most influential predictors included Sentinel-2 Band 8 and Band 12, EVI, PC1, mean77, HH/HV, ARVI, NDVI, RVI, and elevation. Ten-fold cross-validation showed that the CNN-Transformer-CK model had the highest accuracy in predicting forest AGB, with a validation R^2^ of 0.72 and RMSE of 12.18 t/ha, followed by the CNN-Transformer model (R^2^ = 0.69, RMSE = 12.22 t/ha) and RF (R^2^ = 0.59 and RMSE = 14.31 t/ha). The proposed approach supports wall-to-wall AGB mapping for forest management and conservation planning.

## 1. Introduction

Forest aboveground biomass (AGB) is a major component of forest carbon stock and exhibits a strong correlation with the total carbon storage of the ecosystem [1]. Currently, the most accurate AGB estimation method is the traditional field survey, in which structural parameters such as diameter at breast height (DBH) and tree height are measured. While traditional field surveys serve as the gold standard for acquiring comprehensive forest attributes, relying exclusively on them to generate spatially continuous AGB maps over large regions is often constrained by high labor costs and time intensity, especially in complex terrain [2].

Remote sensing, has been a potential alternative for AGB investigations at multiple spatiotemporal scales in recent decades [3]. The mainstream remotely sensed source data used in AGB estimation can be categorized into three types: optical imagery, SAR, and light detection and ranging (LiDAR) [4]. Meanwhile, medium-resolution satellite data such as Sentinel-2 and Landsat are used more commonly among optical remote sensing imagery for forest AGB estimation at the global, national, or regional scale because of their easy accessibility and high spatial match to plot size measured on the ground [5].

However, traditional optical data can only obtain terrain reflectance spectra and provide horizontal information on the forest canopy without canopy penetration capability [6]. Thus, a single optical image is not capable of depicting forest vertical structure and is easily saturated in complex-structure forests, usually leading to an underestimation of high-biomass areas in dense forests [7]. Synthetic aperture radar (SAR) data, particularly at long wavelengths, for example, the L-band, exhibits partial canopy penetration capability, facilitating interactions with arboreal components (branches and foliage). Thus, the returning signal can depict the vertical structure of the forest stand to some extent, lowering the saturation effect of the optical images [8]. Similar to the optical images, the polarized SAR data will be saturated at high AGB values, and the highest saturation level has been found to be approximately 250 t/ha in related studies [9,10]. Therefore, combining optical images with SAR data may further increase the saturation point.

Spaceborne light detection and ranging (LiDAR) systems, including GEDI and ICESat, have become popular in recent studies. However, for example, the laser footprint of the GEDI spans 25 m in diameter and the along-track sampling interval is approximately 60 m, and this discrete sampling cannot provide a wall-to-wall representation of forest attributes [11]. Terrestrial laser scanning (TLS) enables the capture of millimeter-level environmental details and can complement traditional field surveys by providing detailed 3D numerous structural forest inventory attributes [12]. Pre-scan preparations sometimes should be performed before the TLS campaigns, such as the removal of lower tree branches, which is sometimes unacceptable in conservation areas and NFIs [13]. Therefore, combining multi-source remote sensing data, such as the combination of Sentinel-2 and ALOS-PALSAR2, with appropriate modeling algorithms is more practical and promising for AGB estimation [14,15].

The most widely used empirical modeling for AGB estimation can generally be categorized into two types: parametric and non-parametric models. The former require a normal distribution of data involved in the modeling process and cannot deal with complex non-linear relationships among the modeling variables; thus, their usage in practical applications is limited. In contrast, non-parametric models can operate without data distribution specifications; instead, they seek the best-fitting relationship through the properties and structure of the data itself [16]. Popular and commonly used machine learning and deep learning methods for AGB estimation include Random Forest (RF), Support Vector Machine (SVM), Convolutional Neural Network (CNN), Long Short-Term Memory (LSTM), Deep Neural Network (DNN), and Transformer, owing to their superior capability in managing the strong non-linear relationships among variables [17,18]. Research has already proven that by incorporating deep learning techniques, such as CNN and Transformer models, the AGB estimation accuracy can be improved because they can automatically learn spatial features and patterns without relying on hand-crafted features [19,20].

However, these existing non-parametric methods do not consider spatial autocorrelation. These non-parametric models primarily learn the covariate-driven component and may leave spatially structured residual errors due to unobserved environmental controls, disturbance mosaics, and sampling mismatches. Kriging interpolation provides the best linear unbiased prediction (BLUP) of variables based on the theory of variograms and structural analysis and is well suited for minimizing errors in AGB estimation caused by spatial autocorrelation among samples [21]. Therefore, integrating geostatistics with non-parametric models is motivated not only by accuracy improvement but also by the need to characterize the spatial reliability of biomass estimates [22,23]. Guo et al. [24] and Viet Hoang Ho et al. [25] suggested that when spatial autocorrelation is observed in the residuals of machine learning models, it can be fixed by interpolating the residuals of the Kriging method into machine learning predictions.

Above all, this paper proposes a framework of CNN-Transformer with Kriging, comparing the accuracy of these models and selecting the optimal one for Lishui forest AGB mapping. Unlike aggregate statistical means or totals which suffice for regional reporting, spatially explicit AGB maps are indispensable for identifying specific stands requiring silvicultural interventions and defining precise boundaries for biodiversity conservation zones. We expect the results of this study to provide a scientific basis and theoretical support for accurate local accounting of the carbon sequestration value.

## 2. Materials and Methods

### 2.1. Study Area

**Figure 1:**
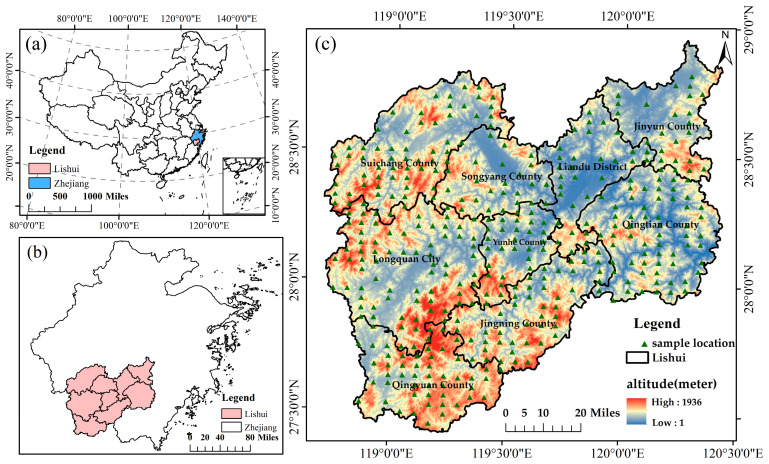
Maps of the study area: (**a**) location of Lishui City, Zhejiang Province; (**b**) location of the study area; and (**c**) topography of the study area and distributions of the forest sample plots. (Map Content Approval Number: GS(2020)4619).

Situated in Zhejiang Province, eastern China, Lishui City occupies a region stretching from 118°41′ E, 27°25′ N to 120°26′ E, 28°57′ N, encompassing approximately 17,300 square kilometers. Administratively, it comprises one municipal district and eight surrounding counties (Figure 1). The topography of the area is dominated by the Wuyi Mountain Range, with elevations varying from just 1 m to 1936 m above sea level, gradually descending from the southwest toward the northeast. Lishui has a central subtropical monsoon climate, characterized by mild winters and warm summers. The average annual temperature hovers around 17.9 °C, dipping to 6.7 °C in January, and peaking at 28.4 °C in July. Rainfall is abundant, with annual precipitation ranging between 1350 mm and 2200 mm, nearly 80% of which falls between March and September. Lishui has abundant natural resources and a superior ecological environment, and is widely known as the largest “natural oxygen bar” in eastern China. The forest coverage rate in Lishui City was 81.7% in 2021. The dominant vegetation type is evergreen forest, and there are diverse mixed forests and rich broad-leaved forests. The dominant tree species include *Pinus massoniana*, *Cunninghamia lanceolata*, *Cinnamomum camphora*, *Pistacia chinensis Bunge* and *Ilex chinensis Sims* [26].

### 2.2. Data Collection, Processing Methods and Variable Extraction

#### 2.2.1. Field Data

The plot data for this study were sourced from the 2020 updated National Forest Inventory (NFI), conducted in the summer of 2014 in the Zhejiang Province. In this inventory, a total of 689 permanent sample plots (size at 0.08 ha (28.28 m × 28.28 m)) were systematically deployed in Lishui city based on the pre-sampling design at the provincial scale. These plots were marked by buried cement piles and revisited every five years under GPS navigation. During the revisit, attributes such as tree species, age, diameter at breast height (DBH), and height (H) of individual trees within each plot were recorded. Ultimately, 398 forest plots remained after removing non-forest plots, which constituted the data basis for subsequent modeling analysis. Our study leveraged species–specific allometric growth equations obtained from the Zhejiang Provincial Center for Forest Resource Monitoring. By plugging in the DBH and H values recorded during the revisit, we calculated the AGB for each tree in our plots. From there, we simply summed the AGB of all trees within a given plot to obtain the total AGB for that forest plot [27,28]. Given that the Sentinel-2 imagery we used had a pixel size of 10 m × 10 m, and our forest sample plots measure 28.28 m × 28.28 m, they were spatially mismatched. To streamline the subsequent modeling and mapping procedures, the value of plot-level (28.28 m × 28.28 m) AGB had to be converted into the value of pixel-level (10 m × 10 m) AGB using per-unit-area values (t/m^2^). Table 1 provides an overview of the statistical characteristics of the AGB values in the final forest plots.

#### 2.2.2. Sentinel-2 Image Pre-Processing and Variables Extraction

Table 2 summarizes the descriptive information of Sentinel-2 images used in this analysis. Of these, Bands 1, 9, and 10 were unsuitable for AGB estimation and were excluded from the analysis. Sentinel-2 Level-2A SR images (GEE code: “COPERNICUS/S2_SR_HARMONIZED”) were downloaded directly from the Google Earth Engine (GEE) platform. The images were further screened using two criteria: (1) a cloud coverage of less than 30% should be satisfied, and (2) the images should be acquired within the time frame (1 June, through 30 September 2020, or the peak growing season of vegetation for this mid-latitude region), and the final selected images were resampled to 10 m resolution and then downloaded to Google Drive for further variable extraction.

Spectral vegetation indices (VIs) may provide more AGB-related information than the individual application of spectral bands [22,29]. Hence, several VIs sensitive to AGB were calculated using the original reflectance bands; the details are summarized in Table 3. Moreover, principal component analysis (PCA) facilitates dimensionality reduction and enhances the interpretability of satellite data [30]. Thus, the first three principal components were considered as the three potential variables for AGB modeling. The first principal component (PC1) of PCA accounted for more than 80% of the original spectral data. PC1 was then leveraged to extract eight image textures using the gray-level co-occurrence matrix (GLCM) method with different window sizes (3 × 3, 5 × 5, and 7 × 7). The resulting texture variables were named by appending the window size to the texture metric; for instance, “mean77” denotes the GLCM mean feature extracted using a 7 × 7 window size. Table 4 illustrates the detailed calculation formulas for GLCM-based texture extraction.

#### 2.2.3. ALOS2-PALSAR2 Data Processing and Variables Extraction

The PALSAR2 imagery was downloaded from the GEE platform and contained backscattering signals of the land surface with dual polarization modes (HH and HV). The downloaded imagery provided by GEE have already been ortho-rectified and slope corrected using the digital surface model ALOS World 3D—30 m. The mosaicked images from 2020 covering the study area were selected and finally resampled to 10 m resolution from 25 m to match the Sentinel-2 images. The radar-related variables used included the HH and HV polarizations, the backscattering ratio HH/HV, the radar forest degradation index (RFDI) (HH − HV)/(HH + HV), and radar RVI (sqrt(HH/(HH + HV)) × (HH/HV)) [37].

#### 2.2.4. DEM Data Pre-Processing and Terrain Variables Extraction

The digital elevation model (DEM) used in this study was mosaicked and extracted using ArcGIS10.8 software from ALOS PALSAR data (available online: https://search.asf.alaska.edu/, accessed on 10 December 2023). The 12.5 m DEM data were resampled to a 10 m resolution as one of the potential variables, and then slope and aspect information were derived as potential variables for subsequent modeling analysis.

### 2.3. AGB Prediction Methods Establishment

In this study, the RF model and CNN-Transformer model coupled with Kriging interpolation analysis were used for AGB mapping. The detailed modeling approaches are summarized as follows:

#### 2.3.1. Random Forest Model

**Figure 2:**
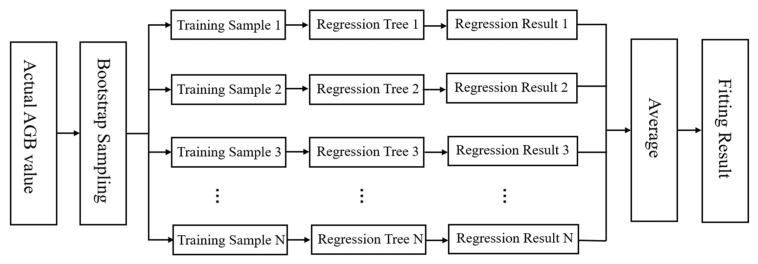
Schematic diagram of Random Forests algorithm for regression analysis.

For regression tasks, RF is adept at handling intricate non-linear interactions between predictors and the dependent variable [38]. The advantages of RF include the ability to easily incorporate or remove predictors based on training data as well as generating variable importance ranking to assess the contribution of individual predictors. Figure 2 shows a schematic diagram of RF-based AGB modeling.

The RF model was carried out using the Random Forest package within the R environment. The package supplies a chart depicting the two key metrics of %IncMSE and IncNodePurity for variable importance ranking. To simplify and reduce computational demands, highly important variables identified by high %IncMSE and IncNodePurity values were selected as predictors for subsequent modeling.

The parameters used in this RF prediction model included ntree, mtry, and node size. where ntree is the count of trained trees, mtry is the quantity of predictor variables chosen at random, and node size denotes the total number of nodes present in the trees. Following several attempts, the values for ntree, mtry, and node size were adjusted to 500, 3, and 10, respectively, in the analysis.

#### 2.3.2. CNN-Transformer Model

**Figure 3:**
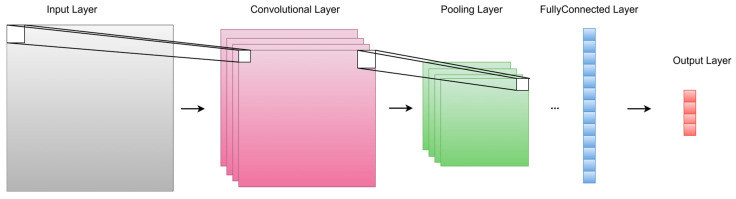
One-dimensional Convolutional Neural Network (1D-CNN) architecture.

CNNs are primarily designed for image processing tasks by applying filters to extract patterns and textures and capture features in images through convolutional layers. The pooling layers in CNNs can further diminish the complexity of feature maps and enhance their resilience to spatial variations. The architecture of a 1D-CNN is shown in Figure 3. The formula of convolution process [39] is as below, (1):

(1) $$h_{\left(/\right)}=ReLU(\omega_{/}*h_{\left(/-1\right)}+b_{\left(/\right)}),\;ReLU(x)=max(0,\;x)$$

where $h_{\left(/\right)}$ represents the feature maps at the layer /, *ReLU* is the activation function, $\omega_{/}$ is the kernel weight, and $b_{\left(/\right)}$ is the bias.

The formula of pooling layer can be represented as below, (2) and (3):

(2) $$g=MaxPool(h_{\left(/\right)})$$

(3) $$m=\left\lfloor \frac{n-p}{s}\right\rfloor +1$$

where g represents the global pooled feature obtained after the pooling operation, p is the window size, n is the input data length, s is the stride length, and m is the output length.

**Figure 4:**
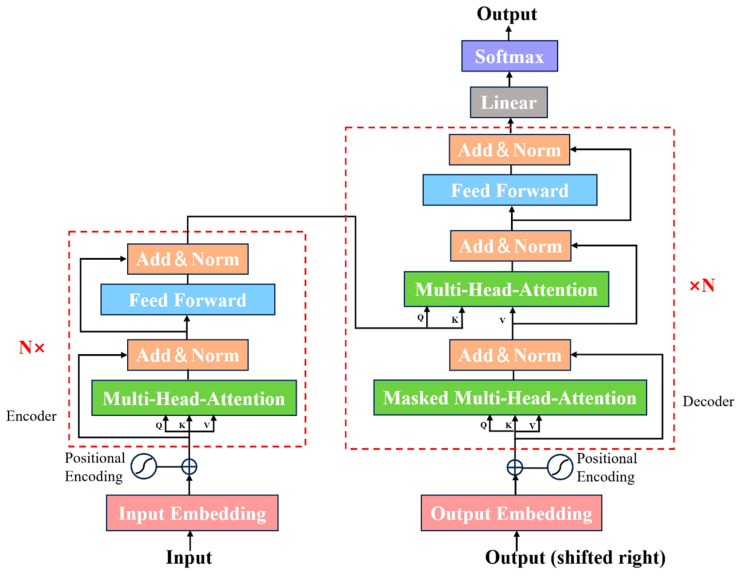
The architecture of Transformer model.

The Transformer mechanism mainly consists of two important components: the encoder and decoder. The encoder includes a multihead attention mechanism, residual connections, and layer normalization, as well as a feed-forward network (FFN). Utilizing multiple heads, the multihead attention system creates various subspaces to concentrate on diverse elements of context information via layer stacking of attention, subsequently relaying this information to the comprehensive connection layer regression output. The decoder of the model has an overall structure similar to that of the encoder, but it incorporates a masking process in its initial multihead attention mechanism. The masking operation restricts the attention mechanism to focusing only on the already generated portions when producing the target sequence, preventing access to the yet-to-be-generated parts [19,40]. This ensures that the target sequence is generated in order and relies solely on information from the previously generated segments. Figure 4 shows the architecture of the Transformer model.

In the Transformer architecture, the self-attention mechanism treats input tokens as an unordered set. Hence, the “positional encodings” must be added to the input embeddings [40]. The formula for positional encoding is as below, (4) and (5):

(4) $$PE\left(p,\;2i\right)=sin(\frac{p}{10000^{\frac{2i}{d}}})$$

(5) $$PE\left(p,\;2i+1\right)=cos(\frac{p}{10000^{\frac{2i}{d}}})$$

where *p* is the position, *i* is the dimension, *PE* is the positional encoding function, and *d* is the dimension of the model. The formula for the self-attention mechanism is as below, (6):

(6) $$Attention\left(V,\;K,\;Q\right)=softmax(\frac{QK^{T}}{\sqrt{d_{k}}})V$$

where $d_{k}$ is the dimension of the key, attention is the attention function, *V* is the value matrix, *K* is the key matrix, *Q* is the query matrix, and softmax is the activation function. The equations for multihead attention are outlined below, (7) and (8).

(7) $$MultiHead\left(Q,\;K,\;V\right)=Concat(h_{1},\cdots ,h_{m})W_{o}$$

(8) $$h_{j}=Attention(W_{j}^{Q}Q,\;W_{j}^{K}K,\;W_{j}^{V}V)\;(j=1,\;\ldots ,\;m)$$

where *m* is the number of self-attention heads; $h_{j}$ is the output of each self-attention; Concat is a function that can merge the outputs of multiple attention heads; $W_{j}^{Q}$, $W_{j}^{K}$, $W_{j}^{V}$ are the parameter matrices of the attention heads of *Q*, *K*, and *V*, respectively; and $W_{o}$ is the parameter matrix after the merging of multiple attention heads.

**Figure 5:**
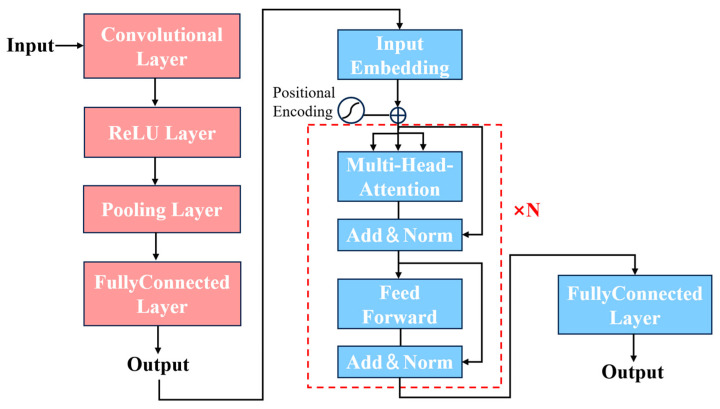
Schematic diagram of CNN-Transformer algorithm established for regression analysis.

Figure 5 shows a conceptual diagram of the CNN-Transformer model established in this research. Here, a CNN-Transformer framework was designed using Python (version 3.9), along with the deep learning tools Pytorch (version 2.3.0) and CUDA (version 12.1), and calculations were executed on the GPU. All analyses were carried out with an Intel(R) Core(TM) i9-14900HX processor, at 2.20 GHz and 16 GB of RAM, and an NVIDIA GeForce RTX 4060 Laptop GPU.

In this study, the selected high-importance variables were used as inputs for the CNN-Transformer modeling. The convolutional layer sets a configuration of 32 filters with a kernel size of 3. ReLu was used as the activation function, and the pooling layer performed max pooling with a pool size of four. Table 5 summarizes the specific hyper-parameters used to train the CNN-Transformer model in the analysis.

#### 2.3.3. Kriging-Based Model

Geostatistical interpolation, as a branch of spatial analysis, has been widely applied across various disciplines. Geostatistics utilizes variogram techniques to quantify spatial dependence structures and derive optimal weights for interpolation [41]. Considering that the residuals obtained from the abovementioned RF and CNN-Transformer models have a level of spatial autocorrelation, the geostatistical method can be used to extract the structurized components hidden in the RF and CNN-Transformer predicted errors (or residuals) of the AGB. Kriging, a geostatistical method, utilizes spatial interpolation in sampled regions to estimate values in other non-sampled regions by using the parameters from the spatial autocorrelation analysis of the residuals. In this study, we used a combination of RF with Kriging and CNN-Transformer with Kriging to determine the spatial distribution of AGB more reliably. Ordinary Kriging (OK) and co-Kriging (CK) were used in this study. Ordinary Kriging relies on one variable to represent the variance of the linear increments, and co-Kriging utilizes not only the primary variable but also cross-correlated secondary variables during interpolation.

In this study, elevation was used as a co-variable for CK interpolation. Cressie proposed the OK and CK formulas [42]. The performance of the semivariogram was evaluated using R^2^ and RMSE. Higher R^2^ values, smaller RMSE, and nugget effects correspond to a better fitting performance.

In the OK and CK modeling approaches, the residual for each sample plot was derived by subtracting the CNN-Transformer-predicted AGB value from the field-observed AGB value, which is defined in Equation (9):

(9) $$Z\left(x_{i}\right)=C\left(x_{i}\right)-{Cˆ}_{CT}(x_{i})$$

where $Z(x_{i})$ is the AGB residual of site *i*, $C\left(x_{i}\right)$ is the field-observed AGB value of site *i*, ${Cˆ}_{CT}(x_{i})$ is the predicted AGB value of site *i* obtained from Transformer model.

The final AGB predictions made by the CNN-Transformer with Kriging were continuously refined in Equation (10):

(10) $${Cˆ}_{CTOK/CTCK}(x_{i})=\;{Cˆ}_{CT}(x_{i})+\;{Zˆ}_{k}(x_{i})$$

where ${Cˆ}_{CTOK/CTCK}(x_{i})$ is the predicted AGB at site *i* using CNN-Transformer-OK or CNN-Transformer-CK, ${Zˆ}_{k}(x_{i})$ is the AGB residual value at site *i*, $C\left(x_{i}\right)$ is the observed AGB at site *i*, ${Cˆ}_{CT}(x_{i})$ is the predicted AGB at site *i* using the CNN-Transformer.

**Figure 6:**
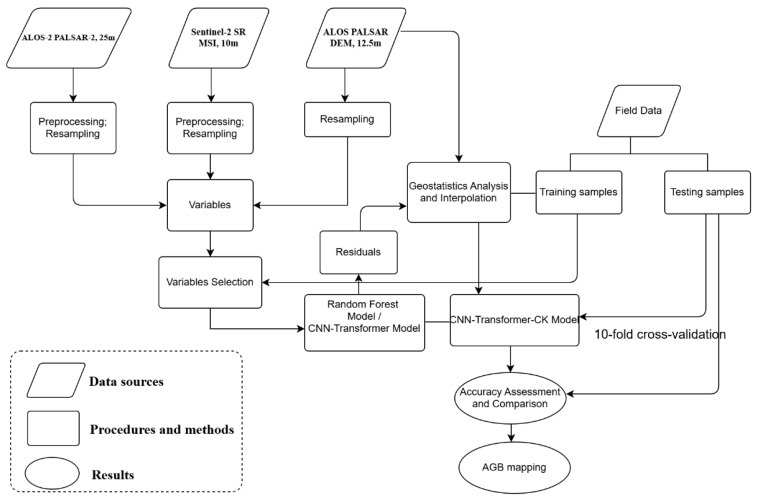
The framework of this study.

Finally, the Sentinel-2 images of study area was classified into four land types using maximum likelihood classification in ENVI 5.6 environment: forest, water, buildings, and cropland. The outcome of the classified forest area served as a refined mask to acquire the forest AGB map of Lishui City. The framework of this study is illustrated in Figure 6.

### 2.4. Accuracy Assessment

This study employed a ten-fold cross-validation method to assess the average predictive accuracy of different models.

A variety of statistical measures, were employed to measure the efficacy of the model, such as the coefficient of determination (R2) in Equation (11), the root mean square error (RMSE) in Equation (12), and the mean absolute error (MAE) in Equation (13), bias in Equation (14), and coefficient of variation (CV) in Equation (15).

(11) $$R^{2}=1-\frac{\sum_{i=1}^{n}{\left(y_{i}-{\hat{y}}_{i}\right)}^{2}}{\sum_{i=1}^{n}{\left(y_{i}-\bar{y}\right)}^{2}}$$

(12) $$\mathrm{RMSE}=\sqrt{\frac{{\sum_{i=1}^{n}\left({\hat{y}}_{i}-y_{i}\right)}^{2}}{n}}$$

(13) $$\mathrm{MAE}=\frac{1}{n}\sum_{i=1}^{n}\left|{\hat{y}}_{i}-y_{i}\right|$$

(14) $$\mathrm{Bias}=\sum_{i=1}^{n}\frac{\left({\hat{y}}_{i}-y_{i}\right)}{n}$$

(15) $$CV\%=\frac{\sqrt{{\sum_{i=1}^{n}\left({\hat{y}}_{i}-y_{i}\right)}^{2}/n}}{\bar{y}}\times 100$$

where *n* represents the quantity of sample plots, ${\hat{y}}_{i}$ is the model-predicted AGB, $y_{i}$ is the field-observed AGB, $\bar{y}$ is the average of all observed AGB.

The relative improvement (RI) index for assessing OK and CK over the CNN-Transformer models was obtained using the following Equation (16):

(16) $$RI=\frac{{RMSE}_{CT}-{RMSE}_{CT-OK/CT-CK}}{{RMSE}_{CT}}$$

## 3. Results

### 3.1. Variable Importance and Selection

**Figure 7:**
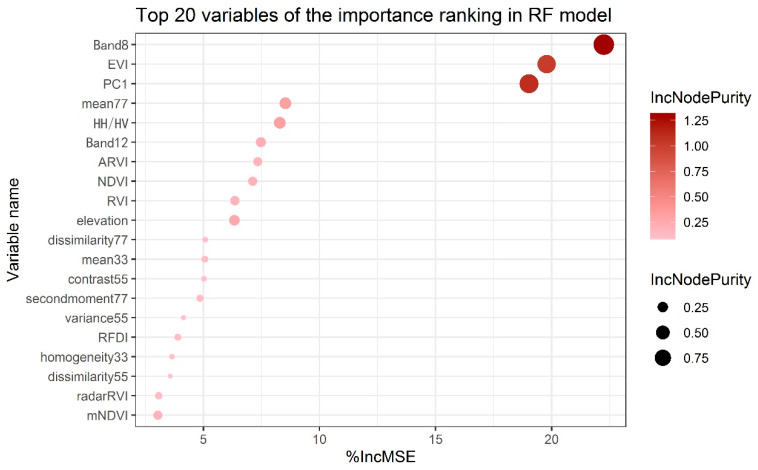
The importance ranking of the predictor variables for AGB in RF models.

In this study, the 10 Sentinel-2 spectral bands, five vegetation indices, the first three principal components of Sentinel-2, 24 GLCM textures with different window sizes from the PC1 image, five ALOS-2 PALSAR-2 polarization features, and three topographic factors (total 50 variables) were used for subsequent variable selection. Figure 7 shows the top 20 variables with high %IncMSE and IncNodePurity values in the RF variable importance analysis, where the size and color of the circles represent the value of IncNodePurity. These two indicators generally reflect the ability of the variables to predict the AGB. Finally, the top 10 variables were selected as subsequent modeling inputs to reduce the computation load and complexity. The 10 variables included Sentinel-2 Band8 and Band12, EVI, PC1, mean77, HH/HV, ARVI, NDVI, RVI, elevation. These 10 variables were employed as inputs for the subsequent RF and CNN-Transformer modeling.

### 3.2. Validation Metrics for the RF and CNN-Transformer Models

Among all 398 sample plots, each fold of the first nine folds used 358 sample plots for model training and 40 for model validation, and the last fold employed 360 sample plots for model training and 38 for validation, with a ten-fold cross-validation method. The performance measures for the two prediction models were obtained. The final R^2^, RMSE, MAE and Bias were calculated as the average value across the ten folds. The training R^2^ of the RF and CNN-Transformer models was 0.94 and 0.85 respectively, while the RMSE was 6.13 and 8.99. The biases of these two models was −0.10 and 0.33, respectively.

As for model validation, Table 6 shows each fold’s validation R^2^ of the RF and CNN-Transformer models utilizing the ten-fold cross-validation approach. The final validation R^2^ of the RF and CNN-Transformer models takes an arithmetic mean of ten folds. Table 7 lists the validation performance and metrics of the two fitted models. The validation R^2^ of the RF and CNN-Transformer models was 0.59 and 0.69 respectively, where 0.69 of CNN-Transformer means the predicted value had a 69% probability to be accepted by this study. The RMSE values of these 2 models were 14.31 and 12.22 t/ha respectively. The MAE values were 10.35 and 9.47 t/ha respectively and the bias values were 0.12 and 0.04 t/ha. Considering the validation metrics of these two models, the CNN-Transformer model had a higher R^2^ and lower RMSE and bias values in the AGB estimation, which means that the CNN-Transformer model performed better in biomass estimation than RF. Hence, the CNN-Transformer model was selected for the subsequent analysis.

### 3.3. Semivariance Analysis of CNN-Transformer-Derived Residuals

**Figure 8:**
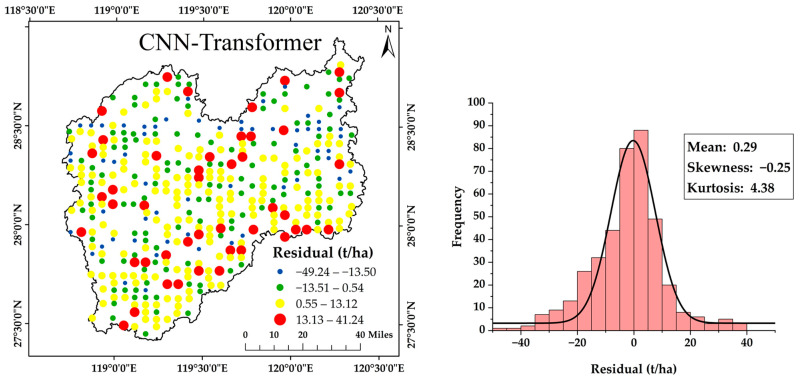
Spatial distribution and frequency histogram of the residuals derived from the CNN-Transformer.

Residuals were derived by subtracting the model-predicted AGB from the ground-measured AGB and collected from each fold of the ten. Table 8 summarizes the statistics of the CNN-Transformer residuals. The mean residual value of the CNN-Transformer was 0.29 t/ha. The residual value range of CNN-Transformer was −49.24–41.24 t/ha and the standard deviation was 12.93 t/ha. The residual values were visualized using several colors and sizes according to their distributions, as shown in Figure 8. The CNN-Transformer residual values were close to the normal distribution, which can be seen from the frequency histogram in Figure 8, and the skewness value of this model was close to 0.

**Figure 9:**
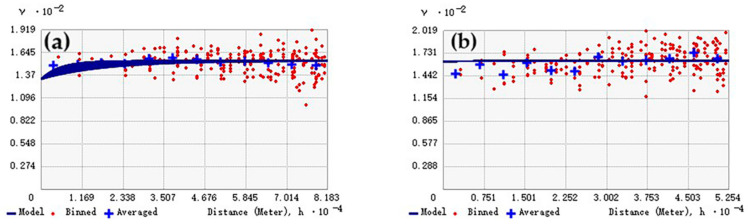
Empirical semivariograms and covariance models for the CNN-Transformer-derived residuals: (**a**) is the semivariogram model of CNN-Transformer using OK analysis; (**b**) is the semivariogram model of CNN-Transformer using CK analysis with a co-variable of elevation. The vertical axis is the 1/2 variance (γ) and covariance of the two positions as the distance increases.

After confirming the approximate normality of the CNN-Transformer model residuals, Ordinary Kriging (OK) and co-Kriging (CK) can be carried out by employing a semivariogram analysis. The model with the smallest RMS and RMSS values closest to 1 was considered as the optimal analytical function. The elevation of the plots was used as a co-variable in the CK interpolation. As shown in Table 9, the exponential model was selected for both OK and CK. Table 9 and Figure 9 show the semivariogram and semivariance models using the OK and CK analyses. Overall, we can see that the CK model had a better fitting performance than the OK model because the former had smaller RMS and nugget/sill values. From the CK models, the smaller nugget/sill value compared with the OK models indicates a stronger spatial correlation by considering elevation as a co-variable. The nugget effect represents micro-scale variability or measurement errors. The range of 35.02 km of the CK model implies that the model residuals are spatially correlated up to this distance, suggesting the presence of regional-scale environmental factors not fully captured by the CNN-Transformer. This also means that the Lishui forest AGB variation was associated with terrain variation.

### 3.4. Forest AGB Mapping Results Based on CNN-Transformer-CK Models

Because the CNN-Transformer model outperformed the RF model, and the CK model performed better than the OK model, this study combined the CNN-Transformer and CK models to investigate their performance as the CNN-Transformer-CK model.

The predicted AGB of the CNN-Transformer-OK and CNN-Transformer-CK models were obtained, and the validation performances and metrics of these two models are shown in Table 10. The R^2^ of these two models was 0.69 and 0.72 while the RMSE was 12.28 and 12.18, respectively. The validation accuracy improvements RI of these two models compared with the original CNN-Transformer are also calculated in Table 10. It can be seen, obviously, that the validation accuracy of the CNN-Transformer-CK was higher than CNN-Transformer-OK. Considering the validation metrics of these two models comprehensively, the CNN-Transformer-CK model was selected for subsequent Lishui City forest AGB mapping.

In Table 10, the R^2^ of the CNN-Transformer-OK (0.69) and CNN-Transformer-CK (0.72) models increased slightly over the original CNN-Transformer model. Although the RI of these two models was only −0.0049 and 0.0032, a slight improvement in accuracy compared with the original model, the OK and CK models considered the spatial heterogeneity of the sample plots. The CK model outperformed the OK model by a small amount based on the RMSE and MAE values.

**Figure 10:**
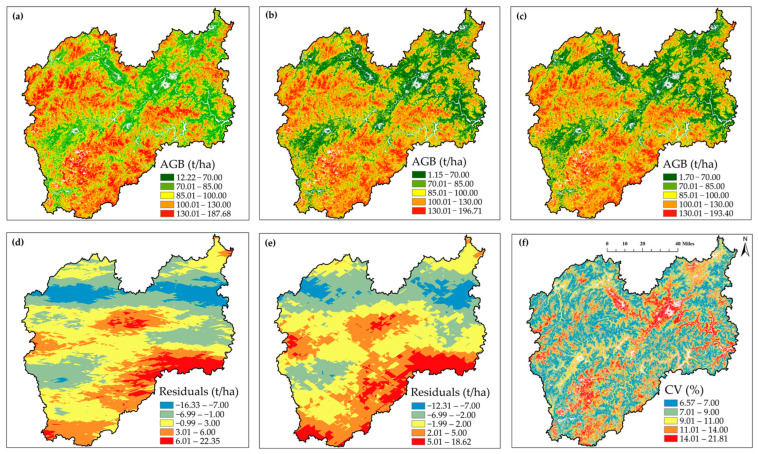
The estimated forest AGB maps obtained from (**a**) CNN-Transformer, (**b**) CNN-Transformer-OK and (**c**) CNN-Transformer-CK; the calculated AGB residuals for (**d**) CNN-Transformer-OK and (**e**) CNN-Transformer-CK models; and the corresponding (**f**) CV map by pixel.

**Figure 11:**
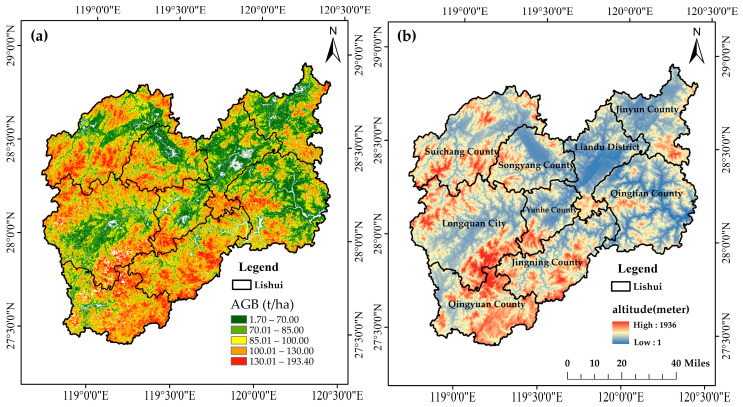
The comparison between (**a**) the estimated forest AGB distribution map from CNN-Transformer-CK model and (**b**) the altitude map in Lishui City.

Additionally, we can see from the prediction map that the model generalization ability is considered. The AGB range reflected by these models can be indicative of the robustness of the models to some extent. The Lishui City forest AGB distribution map obtained from all three models (CNN-Transformer, CNN-Transformer-OK, and CNN-Transformer-CK), the residuals derived from the OK and CK models, and the corresponding CV map by pixel are shown in Figure 10. The predicted AGB range of the CNN-Transformer model was 12.22–187.68 t/ha. The AGB prediction value ranges of the CNN-Transformer-OK and CNN-Transformer-CK models were 1.15–196.71 t/ha and 1.70–193.40 t/ha, respectively. The variation in AGB prediction values between these three models exhibited an improvement in the model generalization ability and AGB prediction reliability when considering spatial variation. The calculated AGB residuals of the CNN-Transformer-OK and CNN-Transformer-CK models range from −16.33 to 22.35 t/ha and −12.31 to 18.62 t/ha, respectively. The coefficient of variation of the predicted AGB distribution map by pixel from the CNN-Transformer model ranged from 6.57% to 21.81%, showing the variation of 10 AGB prediction maps obtained from the CNN-Transformer model using a ten-fold cross-validation approach at the pixel scale. As can be seen from the three AGB prediction maps in Figure 10, the forest areas take up a great part of Lishui City and it deserves to be called the city with the best ecology in eastern China. The northwestern and southern parts of Lishui have relatively higher AGB values than the other regions, while the value distribution trend can also be seen in the altitude map in Figure 11.

The Huang Mao Mountain of 1936 m in Longquan City is the first peak in Lishui City and Zhejiang Province, and is the only natural attraction in southern China, which still maintains a primitive landscape with 200 thousand acres of virgin forests. Although located in the south of China, the terrain of Hang Mao Mountain is similar to that of the Yunnan–Guizhou Plateau, with many natural wonders only in plateaus. However, with the excessive development of tourism, the forest AGB value was relatively lower than that of Jiulong Mountain, Suichang County, because the latter is located in a border area with blocked traffic and light human interference.

## 4. Discussion

### 4.1. Variable Selection

It is important to select the appropriate variables as input parameters for forest AGB estimation before modeling. The extracted variables included four main types: spectral indices, polarization indices, topographic factors, and texture features.

Our work found that the backscattering ratio (HH/HV) of ALOS2-PALSAR2 was highly sensitive to plant growth owing to its associated scattering mechanisms, which are in agreement with the study by Golshani et al. [43]. They demonstrated that ALOS2-PALSAR2 L-band data are sensitive to forest AGB through deep transmissions into large woody branches, trunks, and ground surfaces, and can be used to estimate biomass accurately. Additionally, according to their study, the results also confirmed that SAR data are valuable for forest biomass mapping and should be employed along with optical images, which can be more efficient. Topographic factors (elevation) also played a vital role in this study because hydrothermal conditions varied across different regions. Our study found that the topographic factors developed from ALOS 12.5 DEM had high AGB estimation accuracy, which is consistent with the study by Karabork et al. [44].

In our study, the texture variables (PC1 and mean77) showed great performance in AGB estimation, which is consistent with the studies by Zhou et al. [45] and Li et al. [46]. Our work found that textual extraction from PC1 images can reduce spectral saturation problems and has excellent performance in forest AGB estimation, which is consistent with the results of Su et al. [47]. Zhou et al. [45] pointed out that edge effects exist in the image classification field of textural information, which may reduce the classification accuracy, and the textural information can produce higher accuracy with increasing window size within the threshold. Li et al. mentioned that the texture variables extracted with 7 × 7 window sizes had good performance in AGB estimation in their study area of Hunan Province, and the results of this study align with our findings.

### 4.2. Comparison Between Models

In this study, we first compared the basic RF (R^2^ = 0.59) and CNN-Transformer (R^2^ = 0.69) models for the AGB estimation in Lishui City, Zhejiang Province. From the validation metrics based on ten-fold cross-validation, the latter (CNN-Transformer) was selected for subsequent Kriging interpolation as CNN-Transformer-OK (R^2^ = 0.69) and CNN-Transformer-CK (R^2^ = 0.72). As can be seen in Table 10, the CK model outperformed the OK model and the basic CNN-Transformer model. Hence, the CK model was used as an additional AGB prediction accuracy improvement approach to the basic CNN-Transformer models as CNN-Transformer-CK. Finally, CNN-Transformer-CK was selected for the final Lishui forest AGB distribution map.

From the residuals obtained by each fold of the CNN-Transformer model, there was a trend that the small AGB observations were more likely to be overestimated and the large observations tended to be underestimated. The predicted value (>100 t/ha) obtained by this model was smaller than that of the observed AGB, which is consistent with the results of several studies [48,49,50]. Golshani et al. [43] validated in their study that the saturation point of ALOS2-PALSAR2 was approximately 300 t/ha. Hence, it can be inferred that the saturation problem that occurred in our study may be attributed to optical images. Su et al. [47] and Gao et al. [51] found that the saturation points of optical images range from 15 to 70 t/ha and that the vegetation index can mitigate the saturation problem. Therefore, it can be inferred that in our study, the combination of Sentinel-2 with ALOS-2 PALSAR-2 did not lead to a wide margin of underestimation and controlled the saturation within an acceptable range.

In this study, we found that using Kriging methods (CK and OK) can both slightly improve the prediction accuracy compared with the basic CNN-Transformer model, and it also supplies the gap of spatial correlation. We found that the co-Kriging of the CNN-Transformer model’s residuals performed better than the Ordinary Kriging method. According to this study’s results, the residuals extracted from the CNN-Transformer model did not show very high spatial correlation compared with previous studies, such as Jiang et al. [23] and Chen et al. [52], but using Kriging methods to optimize the prediction accuracy is necessary, similar to the study by Li et al. [7].

This study showed that the distribution of AGB values is highly related to elevation, as shown in Figure 11. The distribution of high AGB values is consistent with high-altitude areas. Previous studies have shown that AGB and elevation have similar trends [53]. The R^2^ and RI of the CK models outperformed those of the OK models in this study, indicating that the employment of elevation in mountainous regions can improve the interpolation accuracy. Additionally, the use of CK models can mitigate the saturation problem in the high AGB value region, which can be seen in Figure 10a,c. By adding the CK model to the original CNN-Transformer model, the maximum AGB value increased and the minimum AGB value declined. Moreover, the relatively small number of sample points (398) in this large-scale study area and sample plots were not completely free from human interference.

Compared with the original deep learning method, CNN-Transformer, which only considers the extracted modeling variables, integrating the co-Kriging method into it takes the spatial autocorrelation into consideration and thus can improve the AGB estimation accuracy. Hence, the employment of the CK model in the original deep learning model is of great importance.

Cai et al. mentioned in their study that by integrating spatial weight, a method of geostatistics, and deep learning techniques, such as CNN-LSTM, the accuracy of NDVI prediction is improved because adding spatial weight is conducive for analyzing spatial nonstationality when it comes to complex spatial relationship modeling [54,55]. A recent study by Maryland University developed a Geo-RF framework to address spatial variability and improve crop classification accuracy [56]. In future research, this new technique can be introduced for biomass estimation to explore its potential in this area.

The sample plots in this study employ a systematic sampling design based on a fixed coordinate grid (typically 4 km × 6 km in this region). In this paper, theoretical semivariogram models of the residuals for CK model analysis revealed a substantial effective range of 35.03 km. This indicates that the spatial autocorrelation of the residuals operates at a broad regional scale (likely driven by macro-topography or climatic gradients) rather than a micro-scale. Given that the sampling design is based on the NFI with an average sampling interval of approximately 6.6 km (derived from 398 plots over 17,300 km^2^), the sampling density is significantly finer than the detected range (sampling interval < range). Therefore, the spatial range supported by our design covers the regional scale. The plot grid is sufficiently dense to reliably capture and model this spatial structure, ensuring that the Kriging interpolation is a valid inference.

The residual correction is most effective in continuous forest areas where residuals exhibit consistent spatial structure. In contrast, in highly fragmented zones or areas with extreme topographic discontinuity, the spatial autocorrelation of residuals may be disrupted. Lishui is characterized by strong elevation gradients and heterogeneous disturbance patterns, which may introduce non-stationarity and abrupt transitions in residuals. Such conditions can violate the intrinsic stationarity and continuity assumptions implicit in the Kriging method, reducing Kriging effectiveness and potentially leading to overly smoothed corrections in fragmented landscapes. Co-Kriging with elevation can partially account for terrain-driven gradients, but it does not fully resolve non-stationarity caused by management and disturbance mosaics. We will explore this issue in our future research.

The hybrid approach allows the deep learning (DL) model to handle the complex non-linear trends, while geostatistics captures the remaining spatially structured errors that the DL model fails to explain. This complementary relationship corrects local biases. We think that the data requirement is a critical constraint. Geostatistical integration strictly requires precise geographic coordinates (X, Y) for every sample plot to calculate the distance matrix and semivariogram.

While the proposed CNN-Transformer-CK model successfully reduces the estimation error for AGB, we recognize that reducing errors for all forest attributes via remote sensing remains a challenge. Remote sensing maps often struggle to capture the full spectrum of detailed information required by inventories, such as specific understory species composition or precise forest health pathology. Therefore, field survey and remote sensing mapping approaches should be viewed as complementary rather than competing.

Traditional field surveys provide high-fidelity data on complex forest attributes that are difficult to invert remotely. In contrast, the wall-to-wall maps generated by our approach bridge the spatial gaps between discrete sample plots. By integrating the high-quality AGB estimates from this study into operational inventories, forest managers can achieve a hybrid monitoring system that combines the detailed accuracy of field plots with the comprehensive spatial coverage of remote sensing maps.

### 4.3. The Effects on Policy

Lishui City endured great human interference between 2008 and 2017, mainly in the southwestern and northeastern parts of Lishui City. According to a study by Xiong et al. [57], these two parts of the city have a large number of residential areas and are more susceptible to landslides, and these areas are experiencing relatively serious forest loss. The areas surrounding densely populated towns, Longquan City, and Suichang County, have a high degree of forest disturbance. These areas have exerted significant pressure and caused considerable damage to forests due to urbanization, industrialization, and transportation infrastructure development. As the most economically developed area in Lishui City, Liandu District has experienced severe forest disturbances in some of its regions.

In order to maintain the forest area and conserve the ecosystem, the Lishui government prepares a restoration project in three years (2021–2023) with a total investment of 5.53 billion RMB. To date, the ecological environment has significantly improved, forest quality has effectively enhanced, biodiversity is increasingly rich, and economic benefits continue to improve. In the past three years, the city’s forest carbon storage has increased by 8%, with a total amount of 62 million tons, ranking first in Zhejiang Province in terms of carbon sequestration capacity. Lishui was selected as one of the first national forestry and carbon sink pilot cities by the National Forestry and Grass Administration.

Beyond the spatial distribution of forest resources, the high-quality AGB estimates achieved in this study hold significant practical value for forest management and policy implementation. Precise biomass quantification is a prerequisite for the accurate accounting of forest carbon sinks. Lishui City is a national pilot city for forestry carbon sinks. In the context of carbon trading and ecological compensation mechanisms, reducing the estimation error directly minimizes the uncertainty in carbon stock calculations. This high confidence level allows for more accurate pricing and trading of carbon credits, ensuring the economic realization of ecological values.

The direct effect of altitude is a key mechanism influencing the biomass of Masson pine forests [58]. Masson pine is the main forest type in Lishui City. The biomass of Masson pine and the Shannon index have indirect interactions [59]. Biomass is also related to canopy density. High forest biomass usually means high species richness, and an increase in species diversity can enhance the overall resource utilization efficiency of a community, thereby improving productivity [60]. Regions with high AGB values demonstrated high species richness. According to the AGB spatial distribution pattern obtained in this study, ecological scientists can obtain valuable information based on this map. Additionally, there is a high chance of intricate ecological interactions in the high AGB value region, which is more suitable for scientific exploration.

Based on the results of this study, the spatial distribution of forest AGB in Lishui City could be effectively predicted using diverse potential predictor variables that were extracted from appropriate image processing technology applied to Sentinel-2 bands, its vegetation indices and textures, ALOS-2 PALSAR-2 polarization features, and topographic factors. Therefore, the findings of this study are beneficial to the scientific advancement of localized forest management strategies and can offer a foundation for regional land-use planning and forest resource management.

Although this study primarily focuses on the status assessment of forest AGB for the year 2020, we recognize that the forest ecosystem is subject to continuous change due to natural growth and anthropogenic disturbances. The high-quality AGB baseline established in this study provides a solid foundation for further research. Moving forward, we are actively extending this work to long-term time-series analysis in Lishui city. We are currently exploring and developing a cascade-based deep learning model designed to effectively enhance change detection.

## 5. Conclusions

In this study, we used Lishui City, Zhejiang Province, as the study area. Based on 398 field forest sample plots, Sentinel-2 multispectral images coupled with ALOS-2 PALSAR-2 and ALOS 12.5 m DEM data were used to extract diverse potential variables to relate to AGB, followed by the selection or identification of those most important variables to model AGB by using Random Forest importance ranking. On this basis, one machine learning model, Random Forest (RF), and one deep learning model, CNN-Transformer, in tandem with two Kriging methods, were applied to model AGB, followed by estimation performance validation using the ten-fold cross-validation approach. Finally, the optimal model for predicting forest AGB was determined to create an AGB map for Lishui City. The main results of this study are as follows:

(1) The spatial distribution of forest AGB in Lishui City could be effectively predicted using diverse potential predictor variables that were extracted from appropriate image processing technology applied to Sentinel-2 bands, its vegetation indexes and textures, ALOS-2 PALSAR-2 polarization features, and topographic factors. The most important predictor variables identified from the Random Forest importance ranking were as follows: Sentinel-2 Band8, Band12, EVI, PC1, mean77, HH/HV, ARVI, NDVI, RVI, and elevation.
(2) The validation results based on the ten-fold cross-validation approach showed that the CNN-Transformer-CK model (with a validation R^2^ = 0.72 and RMSE = 12.18 t/ha) had the highest accuracy in predicting forest AGB in Lishui City by considering the spatial correlation compared with the basic RF (R^2^ = 0.59 and RMSE = 14.31 t/ha) and CNN-Transformer (R^2^ = 0.69 and RMSE = 12.22 t/ha) models.
(3) Combining field survey data with optical images (Sentinel-2), SAR data (ALOS-2 PALSAR-2), deep learning methods, and geostatistical approaches are effective for predicting forest AGB in Lishui City. Hence, the results of this study are helpful for the scientific development of local targeted forest management to some extent, and can also provide a scientific basis for local land-use planning management and forest resource management.

## Figures and Tables

**Table 1:** Statistical summary of the post-filtering plots’ AGB values.

Number of Sample Plots	AGB (t/ha)				Number of Samples Used	
	Range	Median	Average	Std Deviation	Training (Each Fold)	Validation (Each Fold)
398	36.68–184.84	98.78	101.92	22.92	358	40

**Table 2:** Descriptive information of the S-2 images used in the analysis.

Bands Used for Modeling	Central Wavelength (nm)	Spatial Resolution (m)
Band2-Blue	490	10
Band3-Green	560	10
Band4-Red	665	10
Band5-Red Edge	705	20
Band6-Red Edge	740	20
Band7-Red Edge	783	20
Band8-NIR	842	10
Band8A-Narrow NIR	865	20
Band11-SWIR	1610	20
Band12-SWIR	2190	20

**Table 3:** Formula of vegetation indices extracted from S-2 image.

Indices	Reference	Formula
NDVI	[31]	(NIR − RED)/(NIR + RED)
RVI	[32]	NIR/RED
ARVI	[33]	[NIR − (2×RED − BLUE)]/[NIR + (2×RED − BLUE)]
EVI	[34]	2.5(NIR − RED)/(NIR + 6×RED − 7.5×BLUE + 1)
mNDVI	[35]	(NIR − RED)/(NIR + RED − 2×BLUE)

**Table 4:** Gray-level co-occurrence matrix-based textural measures extracted in the current work.

Data	GLCM Texture	Formula	Reference
PC1	Mean	$\sum_{i,j=0}^{N-1}{iP}_{i,j}$	[36]
	Variance	$\sum_{i,j=0}^{N-1}{iP}_{i,j}(1-\mu i)$	
	Homogeneity	$\sum_{i,j=0}^{N-1}{iP}_{i,j}/[1+2(i-j)]$	
	Contrast	$\sum_{i,j=0}^{N-1}{iP}_{i,j}(i-j)$	
	Dissimilarity	$\sum_{i,j=0}^{N-1}{iP}_{i,j}\left|i-j\right|$	
	Entropy	$\sum_{i,j=0}^{N-1}{iP}_{i,j}\ln P_{i,j}$	
	Second moment	$\sum_{i,j=0}^{N-1}i{(P}_{i,j})$ ^2^	
	Correlation	$\sum_{i,j=0}^{N-1}[i(\sum_{i,j=0}^{N-1}ijP_{i,j^{2}}-\mu_{i}\mu_{j})/\sigma_{i}\sigma_{j}]$	

Where *N* is the number of distinct gray levels in the quantized image; *i* and *j* are the two gray levels; $P_{i,j}$ is the joint probability of the gray-level pair (*i*, *j*) in a normalized gray-level spatial dependence matrix; $\mu_{i}$ and $\mu_{j}$ are the means of $P_{i}$ and $P_{j}$, $\sigma_{i}$ and $\sigma_{j}$ are the standard deviations of $P_{i}$ and $P_{j}$.

**Table 5:** Hyper-parameters used to train the CNN-Transformer model.

Model	Hyper-Parameters	Value
CNN-Transformer	Kernel size	3
	Number of filters	32
	Pool size	4
	Number of epochs	200
	Drop rate	0.2
	Learning rate	0.005
	Activation function	ReLu
	Batch size	10
	Number of encoder layers	6
	Number of headers	8

**Table 6:** Each fold’s validation R^2^ of RF and CNN-Transformer models based on the ten-fold cross-validation approach.

Metric	Models	1	2	3	4	5	6	7	8	9	10	Average
**R^2^**	RF	0.3699	0.3888	0.6844	0.6877	0.6023	0.8081	0.8284	0.8081	0.2657	0.4119	0.5855
	CNN-Transformer	0.4166	0.5676	0.7831	0.7766	0.6213	0.8654	0.8592	0.3193	0.795	0.8721	0.6876

**Table 7:** Validation metrics of RF and CNN-Transformer models based on the ten-fold cross-validation approach.

Models	R^2^	RMSE (t/ha)	MAE (t/ha)	Bias (t/ha)
RF	0.59	14.31	10.35	0.12
CNN-Transformer	0.69	12.22	9.47	0.04

**Table 8:** Descriptive statistics of the CNN-Transformer model residuals.

Model	Mean (t/ha)	Std Deviation (t/ha)	Value Range (t/ha)	Skewness	Kurtosis
CNN-Transformer	0.29	12.93	−49.24–41.24	−0.25	4.38

**Table 9:** Parameters of theoretical semivariogram models of the residuals for OK and CK models.

Model Parameter	Theoretical Model	Nugget	Sill	Nugget/Sill	Range(m)	RMS	RMSS
OK	Exponential	132.63	146.41	0.90	54,556.27	12.87	0.98
CK	Exponential	162.78	186.92	0.87	35,026.50	12.87	1.04

**Table 10:** Validation metrics of CNN-Transformer, CNN-Transformer-OK and CNN-Transformer-CK models based on the ten-fold cross-validation approach.

Models	R^2^	RMSE (t/ha)	MAE (t/ha)	Bias (t/ha)	RI
CNN-Transformer	0.69	12.22	9.47	0.04	/
CNN-Transformer-OK	0.69	12.28	9.61	0.13	−0.0049
CNN-Transformer-CK	0.72	12.18	9.52	−0.30	0.0032

## Data Availability

The Sentinel-2 and ALOS2-PALSAR2 data used in this study are openly available in GEE platform accessed on 13 December 2023. The plot data for this study was obtained from Zhejiang Provincial Department of Natural Resources and the data cannot be shared for confidentiality policies. The CNN-Transformer model used and estimated forest AGB maps generated in this study [61] are openly available in the Zenodo database at: https://zenodo.org/records/17349927.
